# The IL-33/IL-31 Axis in Allergic and Immune-Mediated Diseases

**DOI:** 10.3390/ijms24119227

**Published:** 2023-05-25

**Authors:** Giuseppe Murdaca, Sebastiano Gangemi, Monica Greco

**Affiliations:** 1 Department of Internal Medicine, University of Genova and IRCCS Ospedale Policlinico San Martino, 16132 Genova, Italy; 2 School and Operative Unit of Allergy and Clinical Immunology, Department of Clinical and Experimental Medicine, University of Messina, 98125 Messina, Italy; gangemis@unime.it; 3 Internal Medicine Department, San Paolo Hospital, 17100 Savona, Italy; monicagreco89@gmail.com

## 1. Introduction

Interleukin 31 (IL-31) belongs to the IL-6 superfamily. This cytokine is mainly produced by immune cells, such as CD4+ Th2, monocytes/macrophages and dendritic cells, but also in some non-immune cells, such as fibroblasts and keratinocytes. This cytokine plays a role in the pathogenesis of itch, especially in skin conditions such as atopic dermatitis (AD) and psoriasis (Pso) [1].

### The Role of IL-31/IL-33 in Immunopathogenesis

IL-31 also plays a fundamental role in the field of respiratory allergic diseases. Studies conducted on asthmatic patients have demonstrated an increase in plasma IL-31 values and a an inverse correlation with the severity of asthma with lung function. In addition, serum IL-31 levels correlate with the expression level of the IL-31 receptor (IL-31RA and OSMR) in the airways, as well as with Th2-related cytokines (IL-5, IL-13 and TSLP), demonstrating its pro-inflammatory role [2,3]. IL-31 and IL-31RA are upregulated in patients with allergic rhinitis, implying an important role of IL-31 in mucus overproduction, and thus the clinical severity in allergic nasal inflammation [4].

IL-33 belongs to the IL-1 superfamily and is expressed in various cell types in human tissues, as endothelial cells; epithelial cells in barrier tissues; fibroblast reticular cells (FRCs) in lymphoid organs; and glial cells, neurons, and astrocytes in the nervous system [5].

IL-33 sends signals to cells through the membrane-bound ST2 receptor and the IL-33/ST2 axis can enhance the release of pro-inflammatory cytokines in several autoimmune diseases (such as multiple sclerosis (MS), lupus erythematosus (SLE), rheumatoid arthritis (RA), Sjögren’s syndrome (SS), Pso and type I diabetes mellitus (T1DM)) [6].

The role that IL-33/ST2 signaling has in the pathogenesis of autoimmune diseases is driven by its ability to alter the balance between inflammatory Th1/Th17 cells and T regulatory (Treg) cells, promoting the development of autoimmunity. Furthermore, type 2 immune responses depend on regulatory mechanisms of IL-33/ST2 signaling, suggesting an anti-inflammatory implication during autoimmune disorders. Given this, the IL-33/ST2 axis represents a potential biomarker to predict disease severity and activity, as well as the likely efficacy of future clinical treatment [7,8].

As a promoter of the type 2 immune response, IL-33 has also been extensively studied in the pathogenesis of allergic asthma. Indeed, upon its binding to the ST2 receptor, IL-33 stimulates the production of Th2 cytokines, including IL-5, IL-9 and IL-13, by a variety of immune cells [9]. This mechanism appears to be involved in the onset of specific subtypes of asthma, such as childhood asthma, but is also responsible for a higher frequency of exacerbations of the asthmatic disease and the severity of clinical symptoms [10].

From what has been reported above, the correlation between serum and tissue levels of IL-31 and IL-33 in inflammatory disorders has recently been strengthened, which is expressed through a specific association known as the IL-33/IL-31 axis [11].

In fact, researchers have hypothesized that interleukins can stimulate each other, amplifying inflammation and the consequent harmful processes of allergic and autoimmune diseases [11]. In particular, the activation of the Th2/IL-31 immune response involving IL-33/ST2 has a crucial role in the development of allergic inflammation, as in, for example, asthma and allergic rhinitis, where both Th1 responses and Th2 are linked to the expression of IL-31 and IL-33 in asthmatic patients, with a consequent increase in inflammatory cytokines on the bronchi [12].

Murdaca et al. have also highlighted a possible role of the IL-33/IL-31 axis in other immune diseases, such as SLE, RA and systemic sclerosis (SSc), where both cytokines cooperate with synergistic biological mechanisms at the onset and progression of the disease [12].

As reported by Gangemi S. et al. [13] some years ago, IL-31 and IL-33 seem to play a synergistic role in some dermatological diseases, such as AD. They demonstrated the expression of IL-31RA and ST2 on dermal fibroblasts, favoring the synergy between IL-31 and IL-33 on the release of AD-related chemokines from basophils that interact with fibroblasts, in response to EGFR-TK inhibitor therapy. In fact, through the damage to the keratinocytes, the latter favors the release of IL-33, which determines the secretion of various factors capable of causing skin manifestations by binding with its receptor on the mast cells, including IL-31, responsible for itching. Recent studies on the interaction between IL-31 and IL-33 have shown that they also play an essential role in osteoporosis. Indeed, aging affects both Tregs and the IL-31/IL33 axis, resulting in macrophage polarization toward a pro-inflammatory (M1) or anti-inflammatory (M2) phenotype, characterized by specific markers, including found in inflammatory zone 1 (Fizz1). Macrophages that acquire a type 2 immunity-related activation phenotype upregulate IL-33R and express the inflammation-associated protein Fizz1. This imbalance of the IL-31/IL-33 toward a pro-inflammatory form could interfere with the initial immunological responses underlying the onset and progression of osteoporosis [14].

Ferretti E. et al. [15] highlighted the role of the IL-31/IL-31RA axis in cancer, especially in tumors of haematopoietic origin, such as cutaneous T-cell lymphoma (CTLC), follicular lymphoma (FL), mastocytosis and Philadelphia-negative myeloproliferative disease. In the case of FL, for example, IL-31R expression by lymphoid cancer cells has been demonstrated. Furthermore, FL cells exposed to IL-31 were stimulated to proliferate, also due to the expression of IL-4 in the tumor microenvironment.

Moreover, in some solid tumors, such as endometrial carcinoma (EC), the serum levels of IL-31 and IL-33 were found to be significantly elevated and closely correlated with malignancy characteristics, such as the depth of invasion, lymph node involvement and distant metastases.

Given the roles of IL-31 and IL-33 in the pathogenesis of various inflammatory and immune-mediated diseases, as well as some types of cancers, targeting these cytokines would seem a logical choice in the development of new pharmacological targeting agents. This is the case for the humanized monoclonal antibody nemolizumab, which, owing to its IL-31RA blocking action, can reduce the IL-31 cascade and its binding to cutaneous sensory neurons or the downregulation of Th2 inflammatory responses and Th17, therefore modulating inflammation and itch in AD [16].

Regeneron/Sanofi, AstraZeneca, GSK and Genentech/Roche are developing anti-IL-33/ST2 therapies for the management of inflammatory respiratory diseases, such as asthma and chronic obstructive pulmonary disease (COPD). Regeneron’s anti-IL-33 antibody, itepekimab, has been found to be effective in asthma patients, demonstrating that it can reduce the likelihood of asthma control loss by 58% compared to the placebo. Itepekimab, an anti-IL-33 antibody, continues to be developed for COPD, although two phase 3 studies show a non-significant 19% reduction versus placebo in the annual exacerbation rate. Similarly, AstraZeneca’s anti-IL-33 antibody, tozorakimab (MEDI3506), is also currently in phase 2a and phase 3 trials for the treatment of COPD [17].

## 2. Conclusions

As indicated, interleukins IL-31 and IL-33 are at the basis of various pathologies within different organ systems, and fully understanding their pathogenetic role and the different signaling mechanisms will likely lead to new pharmacological treatments based on targeted therapies, and thus better outcomes for our patients.
